# Abiotic Factors Affecting Vector-Borne Plant Pathogen Complexes: Elevated CO_2_ and the Barley Yellow Dwarf Pathosystem

**DOI:** 10.3390/insects16121186

**Published:** 2025-11-22

**Authors:** Shirin Parizad, Jingya Yang, Liesl Oeller, Atoosa Nikoukar, Xi Liang, Arash Rashed

**Affiliations:** 1 Virginia Tech, Southern Piedmont Agricultural Research and Extension Center, Blackstone, VA 23824, USA; shparizad@vt.edu (S.P.); atoosan@vt.edu (A.N.); arashr@vt.edu (A.R.); 2 Department of Plant Sciences, University of Idaho, Parma, ID 83660, USA; 3 Department of Entomology, Plant Pathology and Nematology, University of Idaho, Moscow, ID 83844, USA; elisabeth.oeller@wsu.edu; 4 Department of Entomology, Washington State University, Pullman, WA 99164, USA

**Keywords:** aphid, barley yellow dwarf virus (BYDV), biomass, climate change, elevated carbon dioxide, non-crop hosts, plant–virus–vector interactions, transpiration rate, vector-borne plant virus, virus epidemiology, water-soluble carbohydrate

## Abstract

Changes in atmospheric CO_2_ are known to influence plant physiology, subsequently affecting the nature of their interactions with their biotic environment. Barley yellow dwarf virus (BYDV), one of the most widespread and damaging viruses of small grains, is transmitted by cereal aphids and has a broad range of cultivated and uncultivated hosts from the Poaceae family. Here, we examined the effects of elevated CO_2_ on plant physiology, *Rhopalosiphum padi* L. performance, and the accumulation of BYDV (strain BYDV-PAV) in winter wheat (*Triticum aestivum* L.), foxtail barley (*Hordeum jubatum* L.), and green foxtail (*Setaria viridis* (L.) Beauv.). A growth chamber experiment was conducted under ambient (420 ppm) and elevated CO_2_ (700 ppm) with aphid-infested and uninfested plants. Elevated CO_2_ significantly increased total plant biomass in all species but did not affect aphid survival or reproduction. The root biomass of winter wheat and foxtail barley, but not green foxtail, increased under elevated CO_2_. However, no significant effect of aphids/BYDV was detected on total plant biomass. Transpiration rates varied with host plant and aphid presence but were not affected by CO_2_ level. Total water-soluble carbohydrate concentration was unaffected by CO_2_ or aphids. BYDV-PAV accumulation varied by host plant species, with winter wheat having the highest virus titer, followed by foxtail barley and green foxtail. Virus titers were increased under elevated CO_2_ in all host plant species. We demonstrated that uncultivated grasses are important reservoirs for both BYDV-PAV and the *R. padi* vector and suggested that elevated CO_2_ may enhance virus accumulation across the evaluated host plants. This underscores the need to consider the role of non-crop hosts in developing management plans and/or predicting BYDV dynamics in small grains.

## Simple Summary

This study examined how elevated carbon dioxide (CO_2_) impacts the interactions among host plants, barley yellow dwarf virus (BYDV), and its aphid vector. A growth chamber study was conducted with winter wheat, foxtail barley, and green foxtail as host plants, under ambient and elevated CO_2_ conditions. We measured plant response, aphid performance, and virus accumulation to determine host- and CO_2_-specific effects. Elevated CO_2_ increased total plant biomass across all hosts, but it did not significantly affect bird cherry–oat aphid reproduction or survival. The root biomass of winter wheat and foxtail barley, but not green foxtail, increased under elevated CO_2_. But the presence of aphids/BYDV had no effect on total plant biomass. Transpiration rates varied among host plants and with aphid presence but were unaffected by CO_2_ level. Total water-soluble carbohydrate concentration also differed among host species but did not change with CO_2_ level or aphid feeding. The BYDV concentrations varied among host species, with winter wheat having the highest accumulation of the virus. Virus concentrations increased under elevated CO_2_ in all host plant species. These results indicated that non-crop grasses can function as reservoirs for both BYDV and its aphid vectors, and that elevated CO_2_ may further enhance virus accumulation, potentially contributing to the pathogen spread. Our findings highlight the importance of incorporating non-crop hosts and environmental changes into predictive models of virus outbreaks.

## 1. Introduction

Barley yellow dwarf virus (BYDV) is a phloem-restricted luteovirus in the *Tombusviridae* family that infects major cereal crops such as wheat (*Triticum aestivum* L.), barley (*Hordeum vulgare* L.), and oat (*Avena sativa* L.), among other grassy hosts [1,2,3]. There are several species of BYDV, among which BYDV-PAV is the most prevalent and damaging in the U.S. [4,5]. Barley yellow dwarf virus is transmitted persistently and circulatively—but not propagatively—by cereal aphids; the bird cherry-oat aphid *Rhopalosiphum padi* (Hemiptera: Aphididae) is the primary vector of BYDV-PAV [6,7]. BYDV-infected plants initially exhibit yellowing and/or reddening of leaves. Plants infected at the early stage of development may have stunted growth (hence, the name barley yellow dwarf). A yield loss of up to 30% or higher can occur in high-risk areas [8,9,10].

Although most of the BYDV research to date has been focused on crop fields, the transmission dynamics and the risk of virus spread can be influenced by the surrounding landscape, often dominated by uncultivated grass species [11,12,13]. These non-crop hosts can serve as pathosystem reservoirs since both cereal aphids and BYDV have a vast host range within the Poaceae family [2,14]. Foxtail barley (*Hordeum jubatum* L.), green foxtail (*Setaria viridis* (L.) Beauv.), and downy brome (*Bromus tectorum* L.) are examples of uncultivated grasses that can play a role as reservoirs [2]. However, species-specific differences in transmission efficiency and virus accumulation have also been detected among host plants [2,13]. Different species of cultivated and uncultivated plants are expected to respond differently to biotic and abiotic environmental variables [15,16,17], and this variation in response may differentially impact aphid-BYDV-host plant interactions.

Carbon dioxide (CO_2_) has previously been investigated as an abiotic environmental variable contributing to increases in pest and disease pressure through altering vector distribution and survival, weakening plant defenses, and disrupting interactions between pests and their natural enemies [18,19,20]. The projection of significant increases in CO_2_ levels by the end of the century [21] necessitates studies evaluating the impact of such a change on pathosystems of global significance; BYDV is one of such pathosystems.

Elevated CO_2_ may enhance plant biomass, water-use efficiency, and photosynthetic rates [22,23,24,25,26]. It also alters internal plant chemistry, leading to higher levels of non-structural carbohydrates (e.g., soluble sugars and starch), reduced protein and micronutrient content, and elevated carbon-to-nitrogen (C:N) ratios [27,28,29,30,31]. These shifts can influence virus replication and aphid vector performance in complex, context-dependent ways [32,33]. This is because phloem-feeding insects such as aphids depend on N-rich phloem sap [22,25,29,34].

However, aphid responses to elevated CO_2_ can be variable, with some species exhibiting reduced reproductive rates and delayed development, whereas others benefit from increased sugar concentrations or CO_2_-induced shifts in plant defenses [35,36]. These interactions are often influenced by aphid species, genetic variation in host plants, and host plant nutrient status [22,37,38,39]. Elevated CO_2_ can also directly affect virus–host interactions and has been shown to be associated with increased BYDV-PAV incidence, symptom severity, and virus titers in wheat [33,40]. Recent studies have further shown that BYDV symptoms, such as chlorosis, are more pronounced under combined elevated CO_2_ and temperatures, even in non-crop systems [20]. However, the role of non-crop hosts as reservoirs remains understudied.

We addressed this knowledge gap by quantifying the effects of elevated CO_2_ on two uncultivated species, foxtail barley and green foxtail, as well as a cultivated wheat crop. We first quantified the effects of elevated CO_2_ on biomass, water-soluble carbohydrate concentrations, and transpiration rate and examined whether these effects differ across the three host plant species. Then, we determined whether aphid survivorship and reproduction are differentially influenced by elevated CO_2_ on the three host plant species. Finally, we investigated whether BYDV-PAV titers in host plants are influenced by elevated CO_2_ and whether the effect is species-specific.

## 2. Materials and Methods

### 2.1. Plant and Insect Material

**Plants.** Three plant species were used: winter wheat (*Triticum aestivum* L.; cv. ‘Syngenta Ovation’), a perennial grass foxtail barley (*Hordeum jubatum* L.), and an annual grass green foxtail (*Setaria viridis* (L.) Beauv.). The weed seeds were collected in southcentral and southeastern Idaho by the Weed Management Program at the Aberdeen Research and Extension Center, University of Idaho. The weed seeds were first planted in flat trays in a greenhouse, and after emergence, transplanted into 7.5 cm × 7.5 cm × 20 cm pots, filled with a sandy loam soil collected from the research farm at the Aberdeen Research and Extension Center. Two seeds of winter wheat were planted in pots of 7.5 cm × 7.5 cm × 20 cm and thinned to one after emergence. Greenhouse temperatures ranged between 26 and 30 °C, with a 14 h:10 h (Light[L]:Dark[D]) photoperiod.

**Aphid colonies.** The bird cherry-oat aphid *R. padi* used in this study originated from a colony established by field-collected individuals near Moscow, Idaho, and transferred to the Aberdeen Research and Extension Center in 2013. The aphid colonies were reared separately on barley (cv. ‘Sprinter’) in acrylic cages (20 cm × 25 cm × 40 cm) inside controlled-environment chambers (20–25 °C, 16 h:8 h (L:D)). All aphids used for infestations were viruliferous (BYDV-PAV), and their infection status was regularly confirmed by reverse transcription quantitative polymerase chain reaction (RT-qPCR) (see below).

### 2.2. Experimental Design and Environmental Conditions

A growth chamber experiment was conducted following a split-split plot design to evaluate the effects of CO_2_ on aphid populations and BYDV infection across different host species. The experimental treatments included two CO_2_ levels, ambient (~420 ppm) and elevated (700 ppm); two aphid infestation treatments, aphid-infested and uninfested controls; and three host plant species, winter wheat, foxtail barley, and green foxtail. The CO_2_ level was treated as the whole plot, aphid infestation treatment was the subplot, and host plant species was the sub-subplot. There were 10 plant replicates in each treatment combination, and the study was repeated twice. In the first repeat of the study (2021), the humidity was not controlled (non-operational at the time), but in the second repeat of the study (2023), the relative humidity (RH) was set to 50%.

The potted seedlings (as described in 2.1) were placed inside two growth chambers (FXC-19, BioChambers Inc., Winnipeg, MB, Canada) with elevated or ambient CO_2_ levels depending on the treatment. The photoperiod was set at 16 h:8 h (L:D), and the temperature was 25 °C during the simulated day (L) and 20 °C during the simulated night (D). Approximately two days after plants were placed in the chambers, the CO_2_ treatment was initiated.

Infestations with 2–4 same-sized (~1.5–2 mm), Fully Developed, apterous aphids were conducted seven (replicate 1) and 10 (replicate 2) days following the start of CO_2_ treatments. One 3.8 cm leaf cage (BioQuip, Rancho Dominquez, CA, USA) was placed on one fully developed leaf in each plant for aphid infestation for a 5-day inoculation access period (IAP). After infestation, aphid-infested and uninfested plants were maintained in separate mesh cages (61 cm × 61 cm × 91 cm) in the growth chambers to avoid cross-contamination (hence, the split-split plot design). After five days (i.e., IAP), the leaf was removed along with the cage to eliminate the need for spraying plants. The leaf cages were then removed, and the number of nymphs produced and the remaining adult aphids were counted. Plants inside growth chambers were monitored to ensure complete aphid removal. Aphid mortality rate (adults) was calculated by dividing the number of dead adult aphids by the total number of adult aphids introduced into each cage (i.e., 2–4). Aphid reproduction rate was calculated by dividing the number of nymphs produced during the 5-day IAP by the total number of adult aphids introduced into the cage; nymphs can be readily distinguished from the adults within the first five days.

### 2.3. Transpiration Measurements

Transpiration rate was measured using a portable photosynthesis system (LI-6400, LI-COR Inc., Lincoln, NE, USA) on the newest fully expanded leaf from each pot between 10:00 a.m. and 2:00 p.m., two weeks after aphid removal. Measurements were taken at 26 °C block temperature, 1200 μmol m^−2^ s^−1^ irradiance, and 400 μmol mol^−1^ reference CO_2_ for the ambient CO_2_ level and 700 μmol mol^−1^ for the elevated CO_2_ level.

### 2.4. Plant Biomass and Water-Soluble Carbohydrates Analysis

After four weeks of growth in the growth chambers, all plants were harvested by cutting aboveground biomass at the soil surface, and roots were washed to remove soil. The shoots and roots of individual plants were placed in forced-air drying ovens set to 60 °C until a constant weight was achieved, after which the biomass was weighed.

To measure water-soluble carbohydrates, dried shoot biomass was cut into small sections and ground using a cyclone mill (Cyclone Sample Mill, UDY Corporation, Fort Collins, CO, USA). Approximately 0.2 g of ground shoot biomass was extracted in 10 mL of deionized water by incubating in a water bath at 90 °C for 1 h. Following extraction, samples were allowed to cool to room temperature and then filtered through 0.2-μm cellulose acetate syringe filters to remove particulates. Filtered extracts were analyzed using a high-performance liquid chromatography (HPLC) system (UltiMate 3000, Thermo Fisher Scientific, Waltham, MA, USA) equipped with a High Sensitivity Refractive Index Detector (RefractoMax520, Thermo Fisher Scientific, Waltham, MA, USA). The mobile phase consisted of 0.1% phosphoric acid (HPLC grade, VWR Chemicals BDH, Radnor, PA, USA). The injection volume was set at 50 μL with a constant flow rate of 0.1 mL/min, and the column oven was maintained at 30 °C. The total running time per sample was 26 min. Concentrations of major water-soluble carbohydrate components—glucose, fructose, and sucrose—were determined based on calibration with known standards.

### 2.5. Virus Detection and Quantification

Two weeks after aphid removal, leaf tissue was removed from each plant for RNA extraction, followed by quantitative polymerase chain reaction according to Rashidi et al. [2]. In brief, total RNA was extracted from leaf tissue (~100 mg) using the RNeasy Plant Mini Kit (Qiagen, Hilden, Germany). BYDV copy numbers (titer) were quantified by RT-qPCR using BYDV-PAV–specific primers [41] with the Luna^®^ Universal One-Step RT-qPCR Kit (New England Biolabs, Inc., Ipswich, MA, USA) on a CFX Real-Time PCR System (BioRad Laboratories, Hercules, CA, USA) (repeat 1) and QuantStudio 3 Real-Time PCR System (Thermo Fisher Scientific, Waltham, MA, USA) (repeat 2). Absolute quantification was based on a standard curve generated from plasmid dilutions containing the BYDV-PAV coat protein gene.

### 2.6. Statistical Analysis

All statistical analyses were conducted using full factorial generalized linear mixed models (GLMMs) in SPSS (IBM SPSS Statistics, Version 29). For analyzing plant biomass, carbohydrate concentration, and transpiration rate, fixed effects included CO_2_ level (ambient/elevated), aphid infestation (presence/absence), and host plant species (winter wheat, foxtail barley, or green foxtail). To compare aphid mortality and reproduction, and BYDV-PAV titer, CO_2_ level and host plant species were included as fixed factors. Non-significant interaction items were excluded in a stepwise approach if the effect(s) was largely nonsignificant (*p* ≥ 0.100). Experimental repeat was included as a random factor in all statistical models to control for the variability between the two repeats of the study. Pairwise comparisons were made using Fisher’s least significant difference (LSD) test (α = 0.05).

## 3. Results

### 3.1. Plant Response

**Figure 1:**
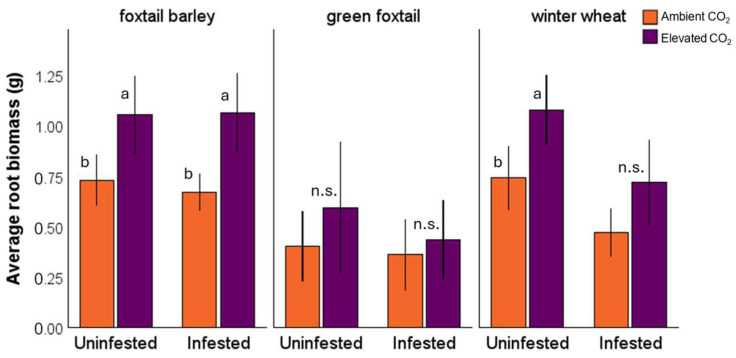
Average root biomass of aphid-infested and uninfested foxtail barley, green foxtail, and winter wheat under ambient and elevated CO_2_. Different letters indicate significant differences between elevated and ambient CO_2_ in aphid-infested and uninfested plants within each host species, and “n.s.” denotes non-significant differences. Error bars represent 95% confidence intervals.

Plant root biomass varied among plant species (*F*_2, 233_ = 24.51, *p* < 0.001), being the highest in foxtail barley and lowest in green foxtail. Overall, root biomass increased under elevated CO_2_ (*F*_1, 233_ = 25.40, *p* < 0.001), with an increasing trend across all three species (Figure 1) (CO_2_ × host plant: *F*_2, 231_ = 1.70, *p* = 0.200). However, this increase was not statistically significant in green foxtail and the winter wheat infested with aphids (Figure 1). Aphid infestation also influenced root biomass (*F*_1, 233_ = 8.20, *p* = 0.005), with a significant reduction detected in winter wheat, but not in foxtail barley or green foxtail.

Total plant biomass aslo increased significantly under elevated CO_2_ (*F*_1, 235_ = 25.81, *p* < 0.001), and varied across host plant species (*F*_2, 235_ = 44.88, *p* < 0.001), with green foxtail (mean ± SE: 7.44 ± 0.58 g/plant) having greater biomass than both winter wheat (4.33 ± 0.15 g/plant; *p* < 0.001) and foxtail barley (4.23 ± 0.13 g/plant; *p* < 0.001). However, unlike root biomass, total host plant biomass was unaffected by aphid/BYDV presence (*F*_1, 235_ = 0.11, *p* = 0.737). There was no significant interactions of aphid × host plant × CO_2_ (*F*_5, 228_ = 0.046, *p* = 0.987), aphid × CO_2_ (*F*_2, 228_ = 0.31, *p* = 0.950), CO_2_ × host plant (*F*_2, 231_ = 0.16, *p* = 0.854), or aphid × host plant (*F*_2, 233_ = 0.31, *p* = 0.735).

**Figure 2:**
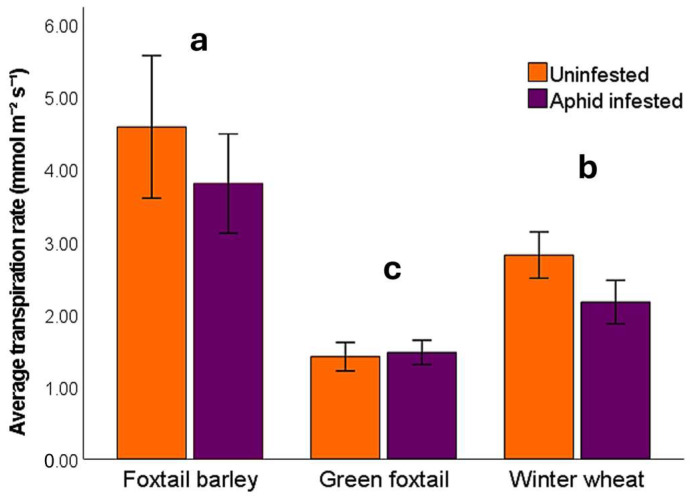
Average transpiration rate in aphid-infested and uninfested foxtail barley, green foxtail, and winter wheat hosts. Different letters indicate significant differences among host plant species. Error bars reflect the 95% confidence intervals.

Transpiration rate differed significantly among host plant species (*F*_2, 215_ = 79.4, *p* < 0.001) and was also overall affected by aphid infestation (*F*_1, 215_ = 7.15, *p* = 0.008), with the exception of green foxtail (Figure 2). Elevated CO_2_ had no detectable effect (*F*_1, 215_ = 0.318, *p* = 0.574) on transpiration rate. Foxtail barley exhibited the highest transpiration rate (mean ± SE: 4.18 ± 0.29 mmol m^−2^ s^−1^), significantly higher than those recorded for winter wheat (2.47 ± 0.11 mmol m^−2^ s^−1^; *p* < 0.001) and green foxtail (1.44 ± 0.06 mmol m^−2^ s^−1^; *p* < 0.001). No significant interactions were detected among aphid presence, CO_2_ level, and plant species (aphid × host plant × CO_2_: *F*_2, 208_ = 0.404, *p* = 0.668; CO_2_ × host plant: *F*_2, 210_ = 0.902, *p* = 0.258; CO_2_ × aphid: *F*_1, 212_ = 1.24, *p* = 0.266; aphid × host plant: *F*_2, 213_ = 2.36, *p* = 0.097).

Total water-soluble carbohydrate concentration differed among host plant species (*F*_2, 228_ = 36.80, *p* < 0.001), but it was not influenced by aphid infestation (*F*_1, 228_ = 2.66, *p* = 0.105) or CO_2_ treatment (*F*_1, 228_ = 0.52, *p* = 0.473). Among host plant species, winter wheat had the highest total carbohydrate concentration (mean ± SE: 71.87 ± 2.92 mg g^−1^), exceeding those of green foxtail (61.24 ± 4.28 mg g^−1^; *p* = 0.001) and foxtail barley (45.28 ± 1.94 mg g^−1^; *p* < 0.001). Additionally, green foxtail also had significantly higher total carbohydrate concentration than foxtail barley (*p* < 0.001), underscoring clear species-specific differences in carbon allocation. Two-way interactions between CO_2_ × host plant (*F*_2, 228_ = 0.49, *p* = 0.611), aphid × host plant (*F*_2, 228_ = 0.57, *p* = 0.568), and aphid × CO_2_ (*F*_1, 228_ = 1.85, *p* = 0.176) were all non-significant and excluded from the final model. The three-way aphid × host plant × CO_2_ interaction was nonsignificant but kept in the final model due to the borderline effect (*F*_7, 228_ = 1.92, *p* = 0.068).

### 3.2. Aphid Response

**Figure 3:**
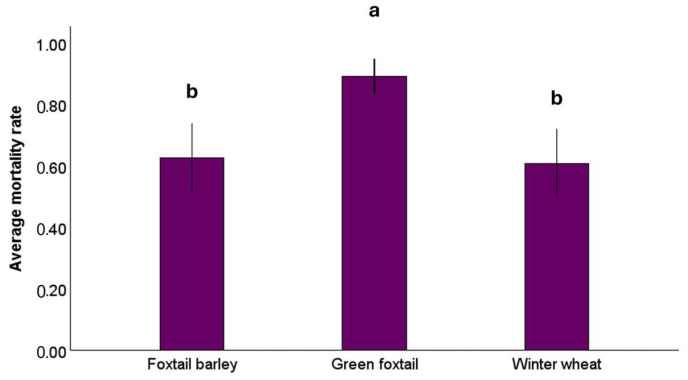
Mean aphid mortality rate of *Rhopalosiphum padi* on foxtail barley, green foxtail, and winter wheat. Different letters indicate statistically significant differences among host plant species (LSD, *p* < 0.05). Error bars reflect the 95% confidence intervals.

Aphid survivorship varied significantly among host plant species (*F*_2, 116_ = 14.66, *p* < 0.001), but the CO_2_ level had no significant effect (*F*_1, 116_ = 0.62, *p* = 0.434) on aphid survival (Figure 3). The mean mortality rate was higher on green foxtail (mean ± SE: 0.89 ± 0.03) compared to winter wheat (0.61 ± 0.05; *p* < 0.001) and foxtail barley (0.63 ± 0.05; *p* < 0.001), whereas no difference was detected in mortality rates between winter wheat and foxtail barley (*p* = 0.749). There was no significant interaction between CO_2_ level and host plant (*F*_2, 114_ = 0.10, *p* = 0.907), and the interaction was excluded from the final model.

**Figure 4:**
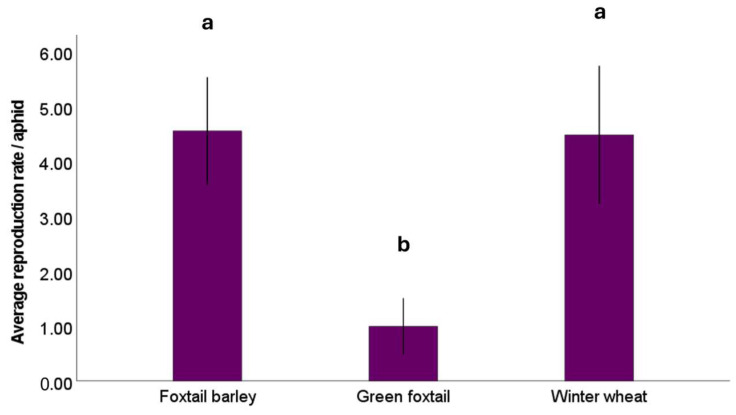
The average number of *Rhopalosiphum padi* nymphs per aphid on each of the three host plants, foxtail barley, green foxtail, and winter wheat. Different letters indicate significant differences among host plant species. Error bars reflect the 95% confidence intervals.

Aphid reproduction also varied significantly with plant species (*F*_2, 116_ = 19.0, *p* < 0.001), but no significant effect of CO_2_ levels (*F*_1, 116_ = 0.81, *p* = 0.370) or CO_2_ × host plant (*F*_2, 114_ = 0.46, *p* = 0.630) was detected (Figure 4). On average, *R. padi* was less reproductive on green foxtail (mean ± SE: 0.99 ± 0.25/aphid) than foxtail barley (4.6 ± 0.48/aphid; *p* < 0.001) and winter wheat (4.5 ± 0.62/aphid; *p* < 0.001). Aphid reproduction did not differ between winter wheat and foxtail barley (*p* = 0.915).

### 3.3. Virus Response

**Figure 5:**
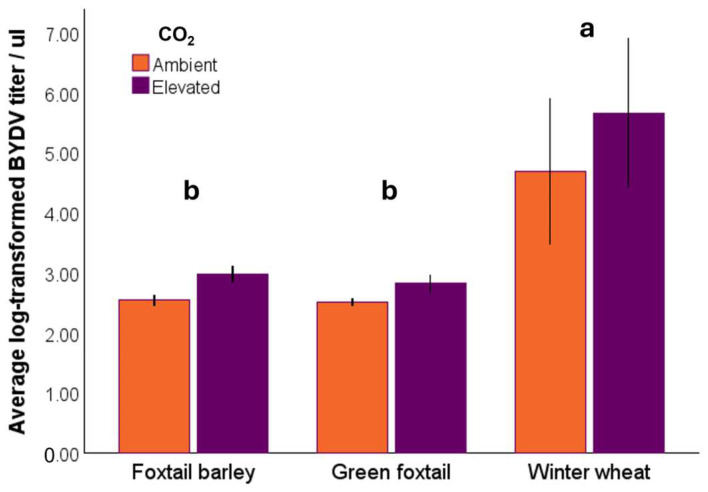
The average log-transformed BYDV-PAV titer/µL extracted from 100 mg of foxtail barley, green foxtail, and winter wheat leaf tissue, under ambient and elevated CO_2_. Different letters indicate significant differences among host plant species. Error bars reflect the 95% confidence intervals.

BYDV-PAV copy numbers (titer) were affected by host plant species (*F*_2, 116_ = 34.41, *p* < 0.001), with winter wheat exhibiting the highest virus accumulation (mean ± SE: 5.16 ± 0.42 copy numbers μL^−1^), followed by foxtail barley (2.75 ± 0.05 copy numbers μL^−1^, *p* < 0.001) and green foxtail (2.66 ± 0.05 copy numbers μL^−1^, *p* < 0.001), whereas the difference between green foxtail and foxtail barley was not significant (*p* = 0.789) (Figure 5). The BYDV titer increased under elevated CO_2_ (*F*_1, 116_ = 4.23, *p* = 0.042). There was no significant interaction between CO_2_ level and plant species (*F*_2, 114_ = 0.53, *p* = 0.587).

## 4. Discussion

We investigated the influence of elevated CO_2_ on plant physiological responses, aphid vector performance, and BYDV accumulation within winter wheat and two uncultivated hosts, green foxtail and foxtail barley. Overall, elevated CO_2_ increased root and total plant biomass across all species, with green foxtail having the highest total plant biomass and the lowest root biomass (Figure 1). This is consistent with previous studies showing that CO_2_ enrichment can enhance crop vegetative growth [23,27]. Root biomass was also negatively affected by aphid infestation; this overall effect, however, was primarily driven by the significant difference in winter wheat (Figure 1). The root biomass reduction in the aphid/BYDV-affected winter wheat was expected as the virus-induced callose deposited in the wheat phloem cells impedes photosynthetic assimilate transport to roots, thereby limiting root growth [42,43]. It is possible that green foxtail and foxtail barley are relatively less susceptible to BYDV-induced phloem blockage or may have mechanisms that limit callose formation and maintain sap transport. The lack of statistical differences between aphid-infested and non-infested uncultivated hosts suggests that green foxtail and foxtail barley may be relatively less susceptible to BYDV- or aphid-induced stress. Future studies examining phloem structure and defense regulation among these hosts could help clarify the physiological basis of this tolerance.

Transpiration rates (Figure 2) were influenced by host plant species and aphid presence, but not by CO_2_ level. Foxtail barley and green foxtail had the highest and the lowest transpiration rates, respectively. Green foxtail is a C_4_ plant, expected to have lower stomatal conductance and transpiration rate than C_3_ species [44], such as wheat and foxtail barley. The observed reduction in transpiration rate in wheat in response to aphid/BYDV presence was also expected, since the reduced root growth (Figure 1) can impair plants’ water and nutrient uptake [32].

Although the total water-soluble carbohydrate concentration varied across host species, it was unaffected by either CO_2_ level or aphid infestation. It was not surprising to observe differences among host plant species since variation in carbohydrate concentration has also been reported at the cultivar level within wheat [45,46]. Our results also suggested that under our experimental setting, the variability in carbohydrate profiles was primarily driven by inherent physiological traits of each host plant species, rather than by aphid herbivory or CO_2_ levels. These findings contrast reports by other studies where elevated CO_2_ increased the accumulation of non-structural carbohydrates, such as soluble sugars and starch [29,31].

Among-species variations in water-soluble carbohydrate concentrations did not explain the observed variations in aphid mortality (Figure 3) and reproduction (Figure 4) on different host plants. *R. padi* had the highest mortality and the lowest rate of reproduction on green foxtail, rendering this plant species a poor host for the vector. In contrast, the highest aphid reproduction occurred on winter wheat and foxtail barley. Moreover, aphid survival and reproduction were not influenced by elevated CO_2_, a finding that contradicts Trębicki et al. [47], who suggested that aphid fecundity may decline in response to increased CO_2_. Inconsistencies across studies indicate that aphid response to CO_2_ may be moderated by an array of host-mediated and biotic and abiotic environmental variables [20,36,48].

The lack of CO_2_ effects on aphid mortality and reproduction likely reflects changes in plant N chemistry rather than carbohydrate dynamics, as our study detected no significant changes in carbohydrate concentrations. Elevated CO_2_ is known to dilute the N content of plant sap [49], raising the C:N ratio and reducing phloem nutritional quality [28,29,49]. Although we did not measure N concentrations directly, it is possible that foliar or phloem N was reduced under elevated CO_2_; reductions in essential amino acids such as histidine, methionine, and leucine can constrain aphid survival and fecundity. Future studies should look more closely at N and amino acid dynamics under elevated CO_2_ to better understand their possible effects on aphids, BYDV, and virus transmission.

Consistent with the observations of aphid reproduction (Figure 4), BYDV accumulation (Figure 5) differed by host plant species, with winter wheat having the highest virus copy numbers, followed by foxtail barley and green foxtail; overall, elevated CO_2_ increased BYDV titers. The increasing pattern of BYDV titers under elevated CO_2_ supports the previous report by Trębicki et al. [40,50]. The BYDV-infected host plants grown under elevated CO_2_ are known to have greater biomass and altered phloem sap composition compared to their counterparts under ambient CO_2_, changes which may improve their palatability to the sap-feeding aphids [33,47], potentially contributing to enhanced virus acquisition and transmission [51].

Our findings complement and further expand those of Rashidi et al. [2], who demonstrated that both green foxtail and foxtail barley can act as sources of BYDV. This study is the first to report that elevated CO_2_ enhances virus titers in uncultivated host plants, potentially contributing to enhanced BYDV spread into cultivated crops. The role of host plant phenology and overwintering potential remains a critical direction for future studies [2].

This study yet again highlighted the importance of non-crop grasses, such as green foxtail and foxtail barley, in the dynamics of the BYDV pathosystem. Although green foxtail was not an ideal host for the aphids or BYDV compared to the other host plants, its robust aboveground growth suggests it could function as a spatial competitor species in grassland systems and contribute to BYDV dynamics. Furthermore, the role of foxtail barley as a perennial reservoir for cereal aphids and BYDV was confirmed under ambient and elevated CO_2_ conditions.

Our findings caution against extrapolating from crop-only systems when predicting virus dynamics, as the interactions among viruses, vectors, and host plants are highly context-dependent and driven by an array of other biological and environmental factors, including, but not limited to, host and vector species, plant developmental stage, and nutrient status. Thus, predictive models of future disease risk must integrate this complexity to more accurately reflect disease dynamics across both managed and natural systems.

While this study focused on CO_2_ as a single abiotic factor, the epidemiology of vector-borne plant viruses is shaped by multiple, interacting biotic and abiotic variables. Among abiotic environmental factors, rising temperatures [20,52,53], elevated ozone (O_3_) levels [54], and drought [55] can influence the BYDV pathosystem through distinct but interrelated pathways affecting host physiology, vector biology and behavior, and virus multiplication. Changes in abiotic environmental factors often do not occur in isolation, and their interaction may shape the pathosystem outcome. For example, when CO_2_ and drought co-occur, their opposing effects on plant defenses and nutrition may balance out, leading to relatively stable aphid performance [56]. Moreover, predictive models suggested that increases in cereal aphid populations due to elevated CO_2_ are expected when N availability is high. However, this effect may be offset when elevated CO_2_ is combined with higher temperatures, resulting in aphid populations similar to those under current conditions [57]. Together, these studies underscore complex interactions among biotic and abiotic variables, the importance of multidisciplinary approaches in investigating the impact of environmental variability on vector-borne pathosystems.

## Data Availability

The original contributions presented in this study are included in the article. Further inquiries can be directed to the corresponding author.
